# Towards Deep Neural Network Models for the Prediction of the Blood–Brain Barrier Permeability for Diverse Organic Compounds

**DOI:** 10.3390/molecules25245901

**Published:** 2020-12-13

**Authors:** Eugene V. Radchenko, Alina S. Dyabina, Vladimir A. Palyulin

**Affiliations:** Department of Chemistry, Lomonosov Moscow State University, 119991 Moscow, Russia; genie@qsar.chem.msu.ru (E.V.R.); aviora@qsar.chem.msu.ru (A.S.D.)

**Keywords:** ADMET, prediction, pharmacokinetics, distribution, blood–brain barrier, permeability

## Abstract

Permeation through the blood–brain barrier (BBB) is among the most important processes controlling the pharmacokinetic properties of drugs and other bioactive compounds. Using the fragmental (substructural) descriptors representing the occurrence number of various substructures, as well as the artificial neural network approach and the double cross-validation procedure, we have developed a predictive in silico *LogBB* model based on an extensive and verified dataset (529 compounds), which is applicable to diverse drugs and drug-like compounds. The model has good predictivity parameters ($Q^{2}=0.815$, $RMSE_{cv}=0.318$) that are similar to or better than those of the most reliable models available in the literature. Larger datasets, and perhaps more sophisticated network architectures, are required to realize the full potential of deep neural networks. The analysis of fragment contributions reveals patterns of influence consistent with the known concepts of structural characteristics that affect the BBB permeability of organic compounds. The external validation of the model confirms good agreement between the predicted and experimental *LogBB* values for most of the compounds. The model enables the evaluation and optimization of the BBB permeability of potential neuroactive agents and other drug compounds.

## 1. Introduction

Permeation through the blood–brain barrier (BBB) is among the most important processes controlling the pharmacokinetic properties of drugs and other bioactive compounds in humans and animals. For compounds targeting the central nervous system (CNS), such a penetration should be enhanced during drug development while, for peripherally acting drugs, it should be avoided to prevent central side effects [1,2]. In addition to the passive diffusion, this process may involve active efflux and uptake transport, as well as the binding of a drug to plasma proteins and brain tissue. In recent years, substantial progress has been made in the development of direct in vivo measurements of the blood-to-brain transport (e.g., microdialysis [3] and cerebral open-flow microperfusion [4]), as well as non-mammalian whole-organism models suitable for the high-throughput screening [5]. Increasingly relevant and accurate in vitro models are also being developed [6,7,8], including cell-free methods such as the widely used parallel artificial membrane permeability assay (PAMPA) or immobilized artificial membrane (IAM) chromatography, brain slices, isolated capillaries, and various cell culture models. Nevertheless, all these approaches obviously require significant amounts of a physical substance, while achieving physiological relevance in a model may be challenging. Thus, the need for robust in silico techniques for the prediction of the BBB permeability of diverse compounds with different transport mechanisms is still quite valid, especially in virtual screening, multiparameter assessment [9], and lead optimization contexts.

Traditionally, the most commonly used quantitative measure of BBB penetration and distribution has been the ratio of total concentrations of a compound in the brain and in plasma or whole blood, usually expressed as a logarithm

(1) $$LogBB=\log\;K_{p}=\log\frac{C_{brain}}{C_{plasma}}$$

Although it is now recognized that this quantity is not the best endpoint for drug optimization because actual bioavailability in the brain can be significantly distorted by non-specific binding on both sides of the barrier [1,3,10], it strongly dominates the body of the available experimental data and is used in the majority of modeling studies. Additional variability limiting the model quality is caused by the time-dependent nature of the distribution process. The reported brain-to-plasma ratios may be based on the concentration or area under curve (AUC) values at different timepoints and in different experimental conditions [11]. In many publications, the more easily accessible and abundant classification data (BBB+ for penetrating and BBB− for non-penetrating compounds), often estimated by the presence or absence of the CNS activity, are used.

Starting with the pioneering works of Levin [12] and Young et al. [13], dozens of papers aiming to predict the BBB permeability were published. A review of earlier results can be found in Garg et al. [14], while the reviews by Raevsky et al. [15], Lanevskij et al. [16], Morales et al. [10], and Liu et al. [17] focus on recent publications. Most of the models are based on some combinations of physico-chemical descriptors, primarily lipophilicity, ionization, molecule size, surface area, polarity, polarizability, and hydrogen bonding ability [18,19,20,21,22,23,24,25,26]. Simple rules of thumb or scores to predict BBB-penetrating compounds, similar to the drug-likeness filters, have been formulated [27,28]. As additional descriptors, orbital and solvation energies calculated by quantum chemistry methods [29], membrane permeation parameters derived from molecular dynamics simulations [30,31,32], and even experimental parameters from chromatography [33,34,35] and ion mobility spectroscopy [36], can be used. External estimates of probability that a compound will undergo active efflux mediated by P‑glycoprotein (P-gp) can also be included [21,24,37]. In other approaches, large pools of various 1D, 2D (including molecular fingerprints), and 3D molecular descriptors calculated by different methods [26,38,39,40,41,42,43,44,45,46,47,48,49,50,51,52] are analyzed by various statistical learning techniques, e.g., multiple linear regression, linear discriminant analysis, partial least squares regression, support vector machines, artificial neural networks, random forests, etc., often in combination with some descriptor selection protocols [23,24,26,43,44,45,46,47,48,49,50,51,52]. However, the limited size of the training sets, use of unverified data, and too-small modeling errors for such an inherently noisy endpoint often give rise to the concerns of possible model overfitting [16].

In view of these issues, the goal of the present work was to develop a predictive in silico blood–brain barrier permeability model, applicable to diverse drugs and drug-like compounds. We decided to focus on the fragmental (substructural) descriptors representing the occurrence number of various substructures. Although rarely employed in the literature for the blood–brain permeability modeling, in combination with artificial neural networks they provide efficient tools for various quantitative structure–property relationship (QSPR) and quantitative structure–activity relationship (QSAR) problems [53,54,55]. Previously, this approach was successfully used by us to model the effects of structure on a number of physico-chemical, pharmacokinetic, and toxicity endpoints such as lipophilicity [56], blood–brain barrier permeability (preliminary model [57]), human intestinal absorption [58], hERG-mediated cardiac toxicity [59], etc. Some of these models are available online at our ADMET Prediction Service page (http://qsar.chem.msu.ru/admet/ accessed 01 November 2020) and have been successfully used to evaluate the key absorption, distribution, metabolism, excretion, toxicity (ADMET) properties of potential drug compounds in the virtual screening and molecular design studies [60,61,62,63]. A secondary goal was to refine the modeling procedures suitable for deep neural networks as well as to evaluate their applicability.

## 2. Results and Discussion

### 2.1. Blood–Brain Barrier Permeability Dataset

**Figure 1:**
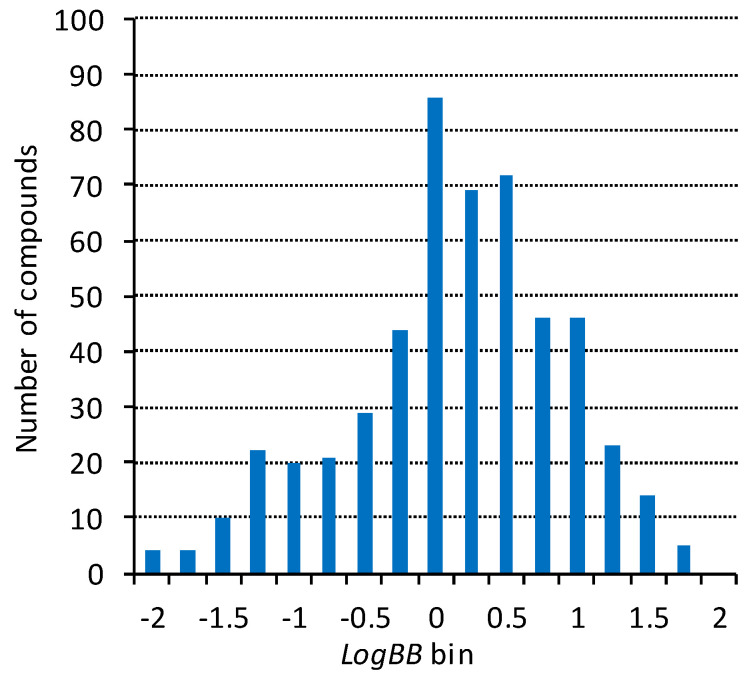
Distribution of the *LogBB* values in the modeling dataset.

Both our experience and the literature data [64,65,66,67] show that the completeness and accuracy of the training sets play a critical role in developing predictive and widely applicable QSAR/QSPR models. We have compiled a dataset based on the open quantitative (*LogBB*) data that was significantly extended and more complete compared to the largest sets published at the time. More than 100 source publications were included. Unfortunately, the quality of the available literature data has not significantly improved in the almost two decades since the analysis [68] was published. During the preparation of the dataset, the data were verified and the errors in structures and endpoint values corrected against the original publications. On the other hand, inorganic molecules irrelevant to medicinal chemistry were excluded. The final dataset used in the modeling contains 529 diverse organic compounds, with *LogBB* values ranging from −2.15 to 1.70 (the full dataset with literature references is provided in the Supplementary Materials). The plot of the *LogBB* value distribution (Figure 1) confirms representative coverage of the entire endpoint range.

Although a large number of papers on *LogBB* modeling have been published, most of them are based on significantly smaller datasets that contain data completely overlapping with our dataset. One of the exceptions is the extensive dataset compiled by Brito-Sánchez et al. [26] which we decided to use as an external validation set. Out of 581 compounds, 13 were excluded because of the unrealistic *LogBB* values (<−2.5). Among the remaining 568 compounds, 216 compounds with *LogBB* values ranging from −2.15 to 1.60 were not present in our dataset. Taking into account the significant number of non-overlapping compounds, instead of merging the data and rebuilding the models, we used this dataset (without any further curation) for additional external “stress-test” validation of the model.

### 2.2. Molecular Descriptors

The fragmental (substructural) descriptors [53,54,55] representing the occurrence number of various substructures were calculated in the framework of the NASAWIN 2.0 [69] software. Linear paths, cycles, and branches were generated using multi-level classification that takes into account atom types, valence states, bonding patterns, and number of attached hydrogens, as well as bond types. The rare fragments that are present in four or fewer compounds, and thus cannot be used to detect general predictive relationships, were removed. The fragments containing up to 10 non-hydrogen atoms were considered in order to provide a sufficiently detailed description of the structures without an excessive increase in the number of descriptors. In total, several thousands of descriptors (depending on the fragment size) were generated.

As sufficiently complete and accurate data on the role of various passive and active transport mechanisms (especially for P-gp substrates) in the transport through the BBB and on the binding to blood plasma proteins and brain tissues are not available for the majority of compounds [1,10], these parameters were not explicitly considered in the modeling. Instead, the related patterns in the effect of structure on the BBB penetration were expected to be modeled implicitly by the neural network-based fragmental model.

### 2.3. Neural Network Modeling Procedure

#### 2.3.1. General Modeling Approach

**Figure 2:**
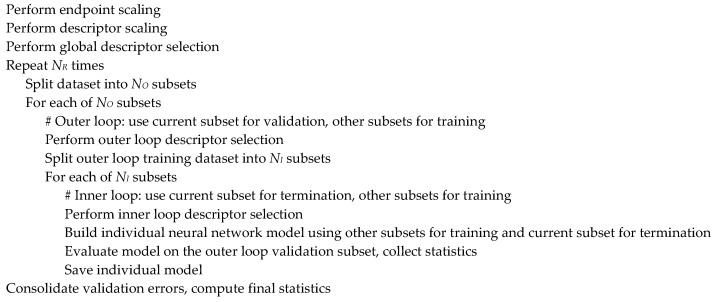
General modeling workflow.

The high-level modeling workflow shown in Figure 2 integrates the classical feed-forward back-propagation neural network (BPNN) architecture and the repeated double cross-validation [70] approach. The double cross-validation procedure involves two loops and in each loop a fraction of the dataset is randomly selected as a test subset. During each iteration of the inner loop, a neural network model is built using the training subset while the prediction error on the test subset is monitored to provide the early termination, while the outer loop test subset is used to validate the resulting model. Usually the 5 × 4-fold double cross-validation scheme is employed, corresponding to $N_{O}=5$ and $N_{I}=4$ in Figure 2. That is, in the outer loop the dataset is split into five subsets of approximately equal sizes, and each of them is used to validate four models built in the inner loop by splitting the remaining data into four subsets of approximately the same size and using three of them for training the model and one for early termination. The procedure can be repeated several times ($N_{R}$) to enhance the stability and reliability of the results. The validation subset errors are then consolidated and normalized into the usual cross-validation statistics such as the $Q^{2}$ parameter and the root mean squared error $RMSE_{cv}$

(2) $$Q^{2}=1-\frac{PRESS}{SS}$$

(3) RMSEcv=PRESSN

where PRESS is the sum of squared prediction errors, SS is the sum of squared deviations from mean, and N is the total number of samples in the dataset. To reduce the risk of overfitting and chance correlations, the inner and outer splits are randomly shuffled at each step. This approach not only provides quite reliable estimates of the model predictivity, but also generates an ensemble of neural network models based on different subsets of data that can be used to improve prediction quality and evaluate the model applicability (see Section 2.3.4). The neural network models were built using the Python script based on the TensorFlow 1.14 and Keras 2.2.4 frameworks on a high-performance NVIDIA GTX1080 GPU.

The neural network architecture may include one or more fully connected (*Dense*) layers. Based on our preliminary testing, the scaled exponential linear unit (SELU) activation function [71] was found to provide the best results in terms of model quality and training efficiency. Optionally, the fully connected layers can be interleaved with the *AlphaDropout* [71] regularization layers in order to prevent overfitting. The mean squared error was used as a loss function for model training.

#### 2.3.2. Data Preprocessing and Descriptor Selection

The preprocessing of raw descriptor and endpoint values should involve some kind of scaling to transform them into finite, small, and consistent intervals suitable for neural network modeling. Several scaler algorithms available in the *scikit-learn* 0.21 framework [72] can be used, including *MinMaxScaler* (transform features by linear scaling to the [0, 1] range), *StandardScaler* (standardize features by removing the mean and scaling to unit variance), *RobustScaler* (outlier-robust standardization by removing the median and scaling to interquartile range), and *QuantileTransformer* (outlier-robust standardization approximating uniform or normal distribution). Each descriptor column is scaled independently.

The descriptors represent integer fragment counts that can vary from zero to several dozens for the types of structures and fragments considered in the modeling. Their distribution is far from normal, and small changes, especially at the lower end of their range, may be significant. Thus, somewhat predictably, in preliminary tests, the *MinMaxScaler* descriptor scaling was found to be superior to the other scalers. Rather unexpectedly, similar results were also obtained for the continuous *LogBB* endpoint values, possibly because the dataset distribution is to some extent skewed. Because of this, in the derivation of final models, the *MinMaxScaler* scaling was used for both the descriptors and the endpoint values.

Descriptor selection is performed globally (for the entire modeling dataset after scaling) as well as locally (for the training sets selected in the outer and inner loops of the double cross-validation procedure). It aims to remove low-variable descriptors (defined as variance below 10^–6^) and to identify the most relevant descriptor subset. For the latter task, three general approaches were implemented on the basis of the *scikit-learn* framework:

- Selection of a specified number of descriptors with the highest F-values in the univariate linear regression (*f_regression*) or non-parametric mutual information scores [73] (*mutual_info_regression*) between the descriptor and the endpoint;
- Recursive feature elimination (RFE) [74] based on the descriptor importance scores from the Partial Least Squares (PLSR), Random Forest, linear Support Vector Machine, ElasticNet or Lasso regression models;
- Stepwise descriptor selection procedure, wherein a multiple linear or Partial Least Squares regression is iteratively refined by adding descriptors with the highest F-value or mutual information scores with the residual endpoint.

Based on preliminary testing, the optimal balance of modeling quality and efficiency is achieved for the PLSR-based stepwise selection procedure using F-value or mutual information scores. Since these models are sufficiently different from the resulting neural network models, we can be reasonably confident that the descriptor selection procedure does not lead to overfitting or chance correlations.

#### 2.3.3. Hyperparameter Optimization

Every machine learning modeling workflow involves a number of hyperparameters that can significantly affect its quality and efficiency. These include model architecture (number and size of the hidden layers) and training parameters, as well as the descriptor set (in particular, fragment size, selection algorithm and the number of selected descriptors) and the prediction and applicability control parameters (Section 2.3.4). In the present study, hyperparameter optimization and model selection were performed using the Hyperopt 0.1.2 [75] library that implements sequential model-based (Bayesian) optimization in the hyperparameter space. The loss function for the minimization was defined as $Loss=-Q^{2}+\frac{\log Time}{100}$ (with time in seconds), aiming to achieve the best model predictivity, preferably in the shortest time. The modeling runs that failed to provide trained models of reasonable quality within specified time limits were discarded, while good models were saved for further analysis. For some of the hyperparameters, the optimal values determined in the preliminary tests were kept fixed during the final modeling.

#### 2.3.4. Prediction and Applicability Control

As mentioned above, the double cross-validation procedure generates an ensemble of neural network models based on different subsets of data that can be used to improve prediction quality and evaluate the model applicability. The prediction procedure involves the following steps:

1. The predicted values are calculated for each individual model in the ensemble and transformed back to the original endpoint scale;
2. For each predicted value, a sanity check is performed to ensure that it lies within a reasonable range ([−2.5, 2.5] for *LogBB*). Values outside of this range (extended compared to the training dataset) most probably indicate that the compound is beyond the model applicability domain limits and the individual predicted value cannot be trusted;
3. If such failed predictions are obtained from more than a specified fraction of the ensemble models (usually 50%), a prediction failure is reported;
4. The individual predicted values are clipped to a specified acceptable range ([−2, 2] for *LogBB*);
5. Mean and standard deviation of the individual predicted values are computed;
6. If the standard deviation is greater than a specified fraction of the acceptable range (usually 30%), a prediction failure is reported;
7. Otherwise, the mean and standard deviation values are reported.

For the analysis and interpretation purposes, the model sensitivities to the descriptors for a particular compound can be evaluated [76] in a local linear approximation as the gradient values (partial derivatives of the scaled network output with respect to scaled inputs) calculated using TensorFlow facilities and averaged over the ensemble models. In order to analyze general trends in the influence of descriptors, these values should be multiplied by the respective scaled inputs and averaged over the prediction set.

### 2.4. Predictive LogBB Model

#### 2.4.1. Optimal Architecture and Model Quality

The preliminary studies of various modeling approaches have shown that the deep neural network architectures containing two or three fully connected hidden layers do not provide significant improvements in model quality for this relatively small training set compared to the shallow (one-layer) networks. On the other hand, they require more training time and increase the risk of overfitting due to greater model complexity. Apparently, larger datasets, and perhaps more sophisticated network architectures, are required to realize the full potential of deep networks.

For this reason, the one-layer network architectures were considered for the final predictive model. During the hyperparameter optimization, three sets of fragmental descriptors were considered, containing up to 6, 8 or 10 non-hydrogen atoms. Descriptor subsets of varying size (from 100 to 1000 descriptors) were selected. The size of a hidden layer relative to the number of descriptors was varied between 0.2 and 0.6, and the dropout layers with probability between 0 and 0.5 were used.

**Figure 3:**
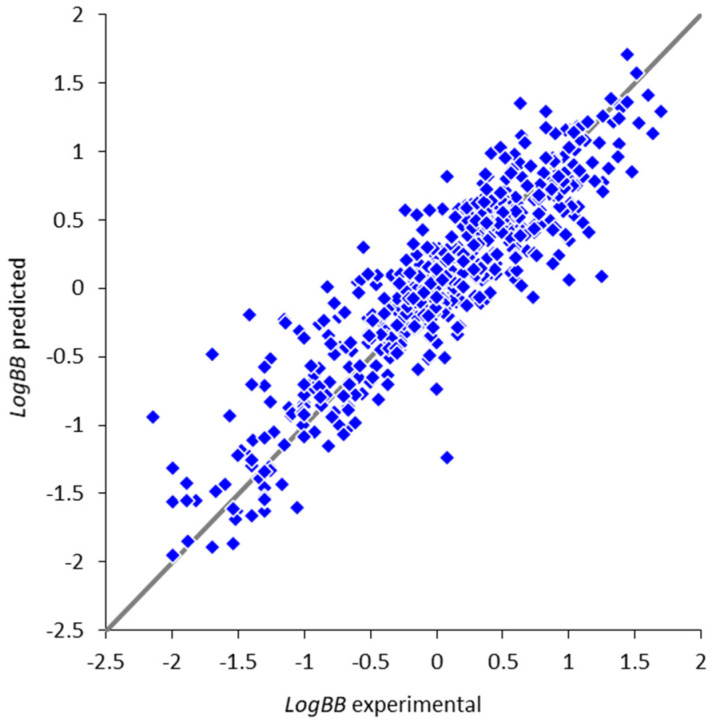
Comparison of the experimental *LogBB* values and the values predicted during double cross-validation.

The optimal model is based on 200 fragmental descriptors containing up to eight non-hydrogen atoms. Its predictivity parameters ($Q^{2}=0.815$, $RMSE_{cv}=0.318$) are similar to or better than those of the most reliable models available in the literature [16,23,26,43,57], and the average prediction error is close to the error of experimental determination of *LogBB* (0.3 log units [27]). The training of all individual neural network models was completed in less than 250 epochs (about 100 epochs for most of them), indicating a low risk of overfitting. The comparison between the experimental *LogBB* values and values predicted during double cross-validation (Figure 3) also confirms high prediction accuracy for the vast majority of compounds. It should be noted that this model is based on a significantly larger and more representative training set, ensuring a broader applicability domain of the model covering more diverse compounds. In addition, the model is indeed able to implicitly handle the peculiarities of the compounds undergoing active influx or efflux: the prediction accuracy for the majority of compounds is quite high, and the significant outliers are not correlated with the known actively transported compounds. The model can provide useful guidance and improve the efficiency of the virtual screening, multiparameter assessment, and lead optimization efforts; however, like any in silico model, its predictions should eventually be validated in vitro and/or in vivo, since a specific compound of interest might be outside of the model applicability domain or could interact with the BBB components (such as transporters and receptors) in some unexpected ways.

#### 2.4.2. Model Interpretation

**Figure 4:**
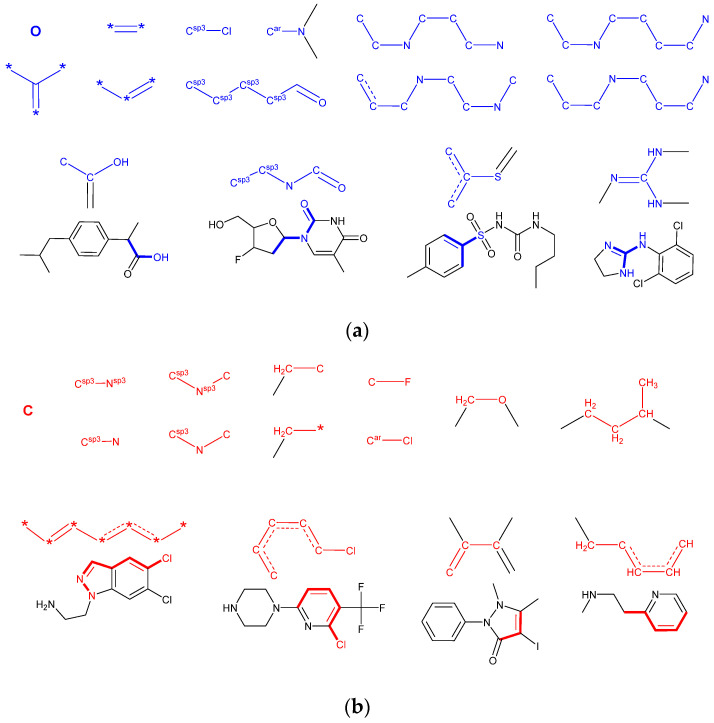
Fragments having the strongest negative (**a**) and positive (**b**) effect on the predicted value of BBB permeability of compounds. Fragments are highlighted in blue for negative and red for positive. Asterisk denotes any atom type; standalone atom symbol means any atom subtype compatible with the specified bond pattern. For more complex fragments, the examples of their occurrence in a structure are shown.

For the analysis and interpretation purposes, the average fragment contributions to the predicted blood–brain permeability over the entire training set were calculated from the model gradient values, as explained in Section 2.3.4. The fragments with the most significant negative and positive influence on the *LogBB* values are shown in Figure 4. Many of them afford a simple interpretation, consistent with the known concepts of structural characteristics that affect the BBB permeability of organic compounds [27,77]. For example, the permeability tends to be higher for carbon-rich aliphatic and aromatic compounds, aliphatic amines, ethers, fluoro-derivatives, and aromatic chloro-derivatives. On the other hand, the presence of oxygen atoms (especially in carboxylic groups), unsaturated groups, amides, polyamines, guanidine derivatives, aliphatic chloro-derivatives, aromatic sulfoxides, sulfones, and sulfonamides tends to decrease the permeability.

Nevertheless, it should be noted that both “positive” and “negative” fragments are usually present and may even overlap in real structures. Thus, their effects may partially compensate each other in subtle non-linear ways. The model also includes a large number of other fragmental descriptors affecting the predicted BBB permeability. Moreover, in contrast to the individual gradient values, the total fragment contributions reveal significant variability between the individual compounds that reflect different numbers of their occurrences in a structure. Thus, in the optimization of the pharmacokinetic properties of a drug, a more detailed visualization approach based on the permeability heatmaps would be helpful, coupled with full-model predictions and virtual screening of proposed structures.

#### 2.4.3. External Validation

**Figure 5:**
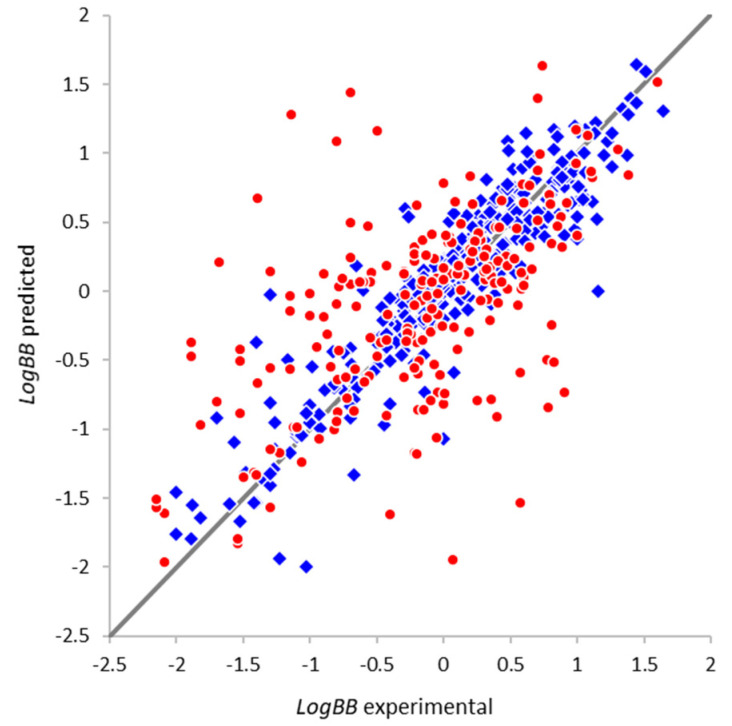
Comparison of the experimental and predicted *LogBB* values for the external validation dataset. The compounds overlapping with our training set are shown as blue diamonds and the non-overlapping compounds are shown as red circles.

As explained in Section 2.1, external validation of the model was performed using the extensive dataset compiled by Brito-Sánchez et al. [26]. Among the 568 compounds with reasonable *LogBB* values, 216 compounds were not present in our training dataset. Their distribution is very similar, with *LogBB* values ranging from −2.15 to 1.60. The prediction using our model was successful for 564 compounds (213 non-overlapping compounds). The prediction results are shown in Figure 5 in terms of the experimental (dataset) and predicted *LogBB* values. It can be seen that the agreement between them is generally good for most of the compounds both in the overlapping and non-overlapping subsets (the statistical parameters are listed in Table 1).

However, the number of outlier compounds with significant errors is greater than desired. Surprisingly, four compounds with absolute errors greater than 1.0 log units were found even among the compounds present in our training set (overlapping subset) while, in our tests, the predictions for the training set using the full ensemble model yield RMSE=0.21 and no errors greater than 0.89. Although the full curation and reconciliation of data was beyond the scope of this study, these compounds strongly exceeded the expected error level and we decided to analyze the possible reasons for this discrepancy. The results are summarized in Table 2. For compounds YG15 and YG16, the *LogBB* values in the validation set, for no obvious reason, do not match the values in the provided reference [78] and the other literature [38] while the training set data seem correct. For tacrine, the training set and most of the literature sources provide *LogBB* values close to −0.12, in agreement with the classical experimental data [79], while the $K_{p}$ value in the referenced source [80] corresponds to LogBB=0.98, still different from the validation set (the values based on the unbound concentrations are in fact close to the commonly accepted value). Finally, for warfarin, the value in the referenced source [81] was calculated from Abraham descriptors rather than determined experimentally. We expect that a more detailed data curation would reveal better concordance between the experimental and predicted values. Nevertheless, this analysis strongly highlights the need for better curation procedures as well as more extensive and representative training data. It should be noted that in the external validation, significant outliers are also not correlated to known actively transported compounds, confirming that the model is able to implicitly handle the peculiarities of the compounds undergoing active influx or efflux.

**Figure 6:**
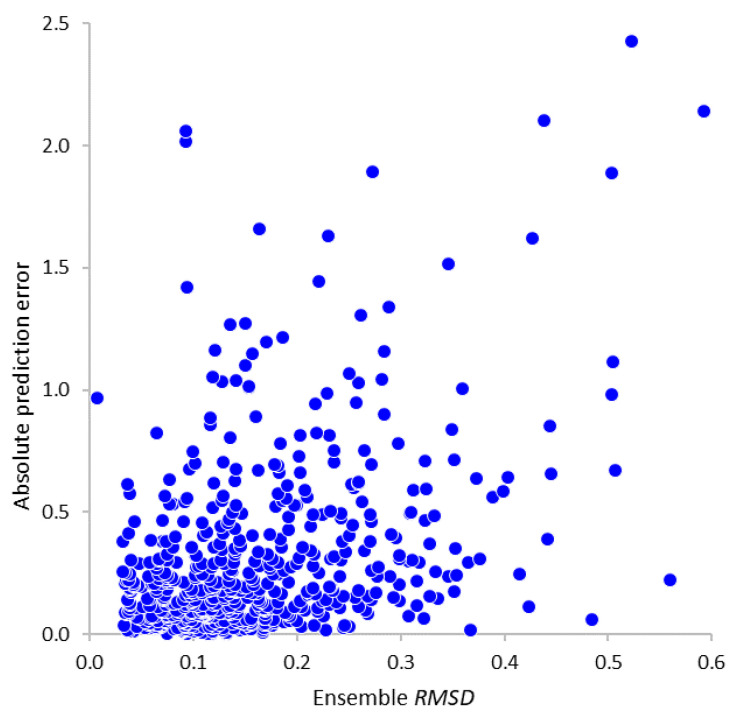
Correlation between the ensemble standard deviations of predicted *LogBB* values and the resulting absolute prediction errors for the external validation dataset compounds.

Using this validation dataset, we also attempted to evaluate to what extent the standard deviation values from the ensemble prediction procedure (Section 2.3.4) could be used as a predictor of resulting prediction errors, and thus as a measure of model applicability. The plot in Figure 6 reveals a loose correlation between these quantities (R=0.40) and indicates that very high prediction errors are indeed much more likely to occur for the compounds with greater ensemble standard deviations (reflecting substantial differences between individual neural network models based on different subsets of training data). However, the accuracy of this test is not sufficient for it to be used as a strict prediction acceptability filter, providing instead just a warning of potential problems.

## 3. Materials and Methods

### 3.1. Blood–Brain Barrier Permeability Datasets

The dataset was compiled from the open quantitative (*LogBB*) data using more than 100 source publications. The data were verified and the errors in structures and endpoint values corrected against the original publications. On the other hand, inorganic molecules irrelevant to medicinal chemistry were excluded. The final dataset used in the modeling contains 529 diverse organic compounds with *LogBB* values ranging from −2.15 to 1.70 (the full dataset with the literature references is provided in the Supplementary Materials).

The external validation dataset was obtained from the publication [26]. Out of 581 compounds, 13 were excluded because of the unrealistic *LogBB* values (<−2.5). No additional data curation was performed.

Instant JChem 20.17 software (ChemAxon Kft., Budapest, Hungary, https://chemaxon.com/) was used for structure database management, search, and analysis.

### 3.2. Modeling Workflow

The fragmental (substructural) descriptors representing the occurrence number of various substructures were calculated in the framework of the NASAWIN 2.0 [69] software. Linear paths, cycles, and branches were generated using multi-level classification that takes into account atom types, valence states, bonding patterns, and number of attached hydrogens, as well as bond types. The rare fragments that are present in four or fewer compounds, and thus cannot be used to detect general predictive relationships, were removed. The fragments containing up to 10 non-hydrogen atoms were considered.

Predictive neural network models were built using the Python script based on the TensorFlow 1.14 and Keras 2.2.4 frameworks on a high-performance NVIDIA GTX1080 GPU. In addition to standard libraries, the *scikit-learn* 0.21 machine learning framework [72] and the Hyperopt 0.1.2 [75] hyperparameter optimization library were used.

## 4. Conclusions

Thus, we have developed a predictive in silico blood–brain barrier permeability (*LogBB*) model based on extensive and verified dataset (529 compounds) and applicable to diverse drugs and drug-like compounds. Using the fragmental (substructural) descriptors representing the occurrence number of various substructures, we have refined the modeling workflow suitable for deep neural networks and evaluated the performance of different options. Playing a key role, the double cross-validation procedure generates an ensemble of neural network models based on different subsets of data that can be used to improve prediction quality and to evaluate the model applicability for a particular compound. It was shown that larger datasets, and perhaps more sophisticated network architectures, are required to realize the full potential of deep neural networks.

Nevertheless, our optimal model has quite good predictivity parameters ($Q^{2}=0.815$, $RMSE_{cv}=0.318$) that are similar to or better than those of the most reliable models available in the literature. In addition, it is based on a significantly larger and more representative training set, ensuring a broader applicability domain of the model covering more diverse compounds. The analysis of the average fragment contributions to the predicted blood–brain permeability reveals influence patterns consistent with the known concepts of structural characteristics that affect the BBB permeability of organic compounds. The external validation of the model on the independent dataset confirms good agreement between the predicted and experimental *LogBB* values for most of the compounds. It was shown that high ensemble standard deviations could provide a warning of potential model applicability problems. The model can provide useful guidance and improve the efficiency of the virtual screening, multiparameter assessment, and lead optimization efforts; however, like any in silico model, its predictions should eventually be validated in vitro and/or in vivo, since a specific compound of interest might be outside of the model applicability domain or could interact with the BBB components in some unexpected ways.

In the future, we plan to extend the blood–brain barrier permeability dataset and make the model available online at our ADMET Prediction Service page (http://qsar.chem.msu.ru/admet/), enabling the evaluation and optimization of BBB permeability and other key ADMET properties of potential neuroactive agents and other drug compounds.

## Figures and Tables

**Table 1:** Statistical parameters for the comparison of experimental and predicted *LogBB* values for the external validation dataset.

Parameter	Full Set	Non-Overlapping Subset
Number of compounds *N*	564	213
Correlation coefficient *R*	0.78	0.58
Root mean squared error *RMSE*	0.47	0.68
Compounds with absolute error > 1.0	33 (6%)	29 (14%)
Compounds with absolute error > 1.5	11 (2%)	11 (5%)

**Table 2:** Additional analysis of some outlier compounds.

Compound	*LogBB* Val ^1^	*LogBB* Train ^2^	*LogBB* Pred ^3^	Notes
2-(2-Aminoethyl)thiazole (YG16)	−1.40 (78)	−0.42	−0.37	Incorrect validation value
2-(2-Dimethylaminoethyl)pyridine (YG15)	−1.30 (131)	−0.06	−0.03	Incorrect validation value
Tacrine	1.16 (146)	−0.13	−0.00	Literature discrepancy
Warfarin	0.00 (520)	−1.30	−1.07	Calculated value in source

^1^ Value in the validation dataset [26], compound number in parentheses. ^2^ Value in our training set. ^3^ Value predicted by our model.

## Supplementary Materials

The following are available online, Modeling dataset and references.
