# Supplementary material for: Towards Deep Neural Network Models for the Prediction of the Blood–Brain Barrier Permeability for Diverse Organic Compounds
Source: Molecules. 2020 Dec 13;25(24):5901. doi: 10.3390/molecules25245901 (PMC7763607; doi:10.3390/molecules25245901)
Supplement: Supplementary file 1 [file molecules-25-05901-s001.zip › LogBBdataset529references.pdf]

Towards Deep Neural Network Models for the Prediction of the Blood–Brain Barrier Permeability for Diverse Organic Compounds

Eugene V. Radchenko, Alina S. Dyabina and Vladimir A. Palyulin *

Supplementary Dataset References

1. Zahlsen, K.; Eide, I.; Nilsen, A.M.; Nilsen, O.G. Inhalation kinetics of C8 to C10 1-alkenes and iso-alkanes in the rat after repeated exposures. *Pharmacol. Toxicol.* **1993**, *73*, 163–168, doi:10.1111/j.1600-0773.1993.tb01557.x.
2. Meulenberg, C.J.; Vijverberg, H.P. Empirical relations predicting human and rat tissue:air partition coefficients of volatile organic compounds. *Toxicol. Appl. Pharmacol.* **2000**, *165*, 206–216, doi:10.1006/taap.2000.8929.
3. Zahlsen, K.; Eide, I.; Nilsen, A.M.; Nilsen, O.G. Inhalation kinetics of C6 to C10 aliphatic, aromatic and naphthenic hydrocarbons in rat after repeated exposures. *Pharmacol. Toxicol.* **1992**, *71*, 144–149, doi:10.1111/j.1600-0773.1992.tb00534.x.
4. Meulenberg, C.J.W.; Wijnker, A.G.; Vijverberg, H.P.M. Relationship between olive oil:air, saline:air, and rat brain:air partition coefficients of organic solvents in vitro. *J. Toxicol. Environ. Health A* **2003**, *66*, 1985–1998, doi:10.1080/713853954.
5. Kaneko, T.; Kim, H.Y.; Wang, P.-Y.; Sato, A. Partition coefficients and hepatic metabolism *in vitro* of 1- and 2-bromopropanes. *J. Occup. Health* **1997**, *39*, 341–342, doi:https://doi.org/10.1539/joh.39.341.
6. Filser, J.G.; Schmidbauer, R.; Rampf, F.; Baur, C.M.; Pütz, C.; Csanády, G.A. Toxicokinetics of inhaled propylene in mouse, rat, and human. *Toxicol. Appl. Pharmacol.* **2000**, *169*, 40–51, doi:10.1006/taap.2000.9027.
7. Sweeney, L.M.; Himmelstein, M.W.; Gargas, M.L. Development of a preliminary physiologically based toxicokinetic (PBTK) model for 1,3-butadiene risk assessment. *Chem.-Biol. Interact.* **2001**, *135–136*, 303–322, doi:10.1016/s0009-2797(01)00177-6.
8. Fiserova-Bergerova, V.; Diaz, M.L. Determination and prediction of tissue-gas partition coefficients. *Int. Arch. Occup. Environ. Health* **1986**, *58*, 75–87, doi:10.1007/BF00378543.
9. Kaneko, T.; Wang, P.-Y.; Sato, A. Partition coefficients for gasoline additives and their metabolites. *J. Occup. Health* **2000**, *42*, 86–87, doi:https://doi.org/10.1539/joh.42.86.
10. Krishnan, K.; Gargas, M.L.; Fennell, T.R.; Andersen, M.E. A physiologically based description of ethylene oxide dosimetry in the rat. *Toxicol. Ind. Health* **1992**, *8*, 121–140, doi:10.1177/074823379200800301.
11. James, B.K.; Ladd, W.S.; Richard, D.F.; James, R.O.; Paul, E.N. In vitro hepatic metabolism of PCBTF: Development of Vmax and Km values and partition coefficients and their use in an inhalation PBPK model. *Inhal. Toxicol.* **1998**, *10*, 65–85, doi:10.1080/089583798197880.
12. Dix, K.J.; Coleman, D.P.; Fossett, J.E.; Gaudette, N.F.; Stanley, A.P.; Thomas, B.F.; Jeffcoat, A.R. Disposition of propargyl alcohol in rat and mouse after intravenous, oral, dermal and inhalation exposure. *Xenobiotica* **2001**, *31*, 357–375, doi:10.1080/00498250110053228.
13. Teo, S.K.; Kedderis, G.L.; Gargas, M.L. Determination of tissue partition coefficients for volatile tissue-reactive chemicals: acrylonitrile and its metabolite 2-cyanoethylene oxide. *Toxicol. Appl. Pharmacol.* **1994**, *128*, 92–96, doi:10.1006/taap.1994.1184.
14. Yamamoto, F.; Oka, H.; Antoku, S.; Ichiya, Y.; Masuda, K.; Maeda, M. Synthesis and characterization of lipophilic 1-[18F]fluoroalkyl-2-nitroimidazoles for imaging hypoxia. *Biol. Pharm. Bull.* **1999**, *22*, 590–597, doi:10.1248/bpb.22.590.
15. Seimbille, Y.; Bénard, F.; Rousseau, J.; Pepin, E.; Aliaga, A.; Tessier, G.; van Lier, J.E. Impact on estrogen receptor binding and target tissue uptake of [18F]fluorine substitution at the 16alpha-position of fulvestrant (faslodex; ICI 182,780). *Nucl. Med. Biol.* **2004**, *31*, 691–698, doi:10.1016/j.nucmedbio.2004.02.010.
16. Hanson, R.N.; Hassan, M. Phenylpiperazine-based radiopharmaceuticals for brain imaging. 3. Synthesis and evaluation of radioiodinated 1-alkyl-4-phenylpiperazines. *J. Med. Chem.* **1987**, *30*, 29–34, doi:10.1021/jm00384a005.
17. Arendt, R.M.; Greenblatt, D.J.; Liebisch, D.C.; Luu, M.D.; Paul, S.M. Determinants of benzodiazepine brain uptake: lipophilicity versus binding affinity. *Psychopharmacology (Berl.)* **1987**, *93*, 72–76, doi:10.1007/BF02439589.
18. Sako, K.; Diksic, M.; Kato, A.; Yamamoto, Y.L.; Feindel, W. Evaluation of [18F]-4-fluoroantipyrine as a new blood flow tracer for multiradionuclide autoradiography. *J. Cereb. Blood Flow Metab.* **1984**, *4*, 259–263, doi:10.1038/jcbfm.1984.35.
19. Tramposch, K.M.; Kung, H.F.; Blau, M. Synthesis and tissue distribution study of iodine-labeled benzyl- and xylylamines. *J. Med. Chem.* **1982**, *25*, 870–873, doi:10.1021/jm00349a022.
20. Blakey, G.E.; Nestorov, I.A.; Arundel, P.A.; Aarons, L.J.; Rowland, M. Quantitative structure-pharmacokinetics relationships: I. Development of a whole-body physiologically based model to characterize changes in pharmacokinetics across a homologous series of barbiturates in the rat. *J. Pharmacokinet. Biopharm.* **1997**, *25*, 277–312, doi:10.1023/a:1025771608474.
21. Kelder, J.; Grootenhuis, P.D.; Bayada, D.M.; Delbressine, L.P.; Ploemen, J.P. Polar molecular surface as a dominating determinant for oral absorption and brain penetration of drugs. *Pharm. Res.* **1999**, *16*, 1514–1519, doi:10.1023/a:1015040217741.
22. Zhu, Z.; Guo, N.; Narendran, R.; Erritzoe, D.; Ekelund, J.; Hwang, D.-R.; Bae, S.-A.; Laruelle, M.; Huang, Y. The new PET imaging agent [11C]AFE is a selective serotonin transporter ligand with fast brain uptake kinetics. *Nucl. Med. Biol.* **2004**, *31*, 983–994, doi:10.1016/j.nucmedbio.2004.07.003.
23. Han, K.S.; Kim, Y.G.; Yoo, J.K.; Lee, J.W.; Lee, M.G. Pharmacokinetics of a new reversible proton pump inhibitor, YH1885, after intravenous and oral administrations to rats and dogs: hepatic first-pass effect in rats. *Biopharm. Drug Dispos.* **1998**, *19*, 493–500, doi:10.1002/(sici)1099-081x(1998110)19:8<493::aid-bdd129>3.0.co;2-z.

24. Lapka, R. Pharmacokinetics and brain entry of alaptide, a novel nootropic agent, in mice, rats and rabbits. *J. Pharm. Pharmacol.* **1991**, *43*, 874–876, doi:10.1111/j.2042-7158.1991.tb03200.x.
25. Ståhle, L.; Guzenda, E.; Ljungdahl-Ståhle, E. Pharmacokinetics and extracellular distribution to blood, brain, and muscle of alovudine (3′-fluorothymidine) and zidovudine in the rat studied by microdialysis. *J. Acquir. Immune Defic. Syndr.* **1993**, *6*, 435–439.
26. Cheng, H.Y.; Liu, T.; Feuerstein, G.; Barone, F.C. Distribution of spin-trapping compounds in rat blood and brain: in vivo microdialysis determination. *Free Radic. Biol. Med.* **1993**, *14*, 243–250, doi:10.1016/0891-5849(93)90021-l.
27. Okuyama, S.; Aihara, H. The mode of action of analgesic drugs in adjuvant arthritic rats as an experimental model of chronic inflammatory pain: possible central analgesic action of acidic nonsteroidal antiinflammatory drugs. *Jpn. J. Pharmacol.* **1984**, *35*, 95–103, doi:10.1254/jjp.35.95.
28. Bolander, H.G.; Wahlström, G.; Norberg, L. Reevaluation of potency and pharmacokinetic properties of some lipid-soluble barbiturates with an EEG-threshold method. *Acta Pharmacol. Toxicol. (Copenh.)* **1984**, *54*, 33–40, doi:10.1111/j.1600-0773.1984.tb01892.x.
29. Subramanian, G.; Kitchen, D.B. Computational models to predict blood-brain barrier permeation and CNS activity. *J. Comput.-Aided Mol. Des.* **2003**, *17*, 643–664, doi:10.1023/b:jcam.0000017372.32162.37.
30. de Lange, E.C.; Danhof, M.; de Boer, A.G.; Breimer, D.D. Critical factors of intracerebral microdialysis as a technique to determine the pharmacokinetics of drugs in rat brain. *Brain Res.* **1994**, *666*, 1–8, doi:10.1016/0006-8993(94)90276-3.
31. Moerlein, S.M.; Laufer, P.; Stöcklin, G. Effect of lipophilicity on the in vivo localization of radiolabelled spiperone analogues. *Int. J. Nucl. Med. Biol.* **1985**, *12*, 353–356, doi:10.1016/s0047-0740(85)80003-6.
32. Abraham, M.H.; Chadha, H.S.; Mitchell, R.C. Hydrogen-bonding. Part 36. Determination of blood brain distribution using octanol-water partition coefficients. *Drug Des. Discov.* **1995**, *13*, 123–131.
33. Björkman, S. Prediction of the volume of distribution of a drug: which tissue-plasma partition coefficients are needed? *J. Pharm. Pharmacol.* **2002**, *54*, 1237–1245, doi:10.1211/002235702320402080.
34. Yoo, S.D.; Shin, B.S.; Kwack, S.J.; Lee, B.M.; Park, K.L.; Han, S.Y.; Kim, H.S. Pharmacokinetic disposition and tissue distribution of bisphenol A in rats after intravenous administration. *J. Toxicol. Environ. Health A* **2000**, *61*, 131–139, doi:10.1080/00984100050120415.
35. Nakazono, T.; Murakami, T.; Higashi, Y.; Yata, N. Study on brain uptake of local anesthetics in rats. *J Pharmacobiodyn* **1991**, *14*, 605–613, doi:10.1248/bpb1978.14.605.
36. Salminen, T.; Pulli, A.; Taskinen, J. Relationship between immobilised artificial membrane chromatographic retention and the brain penetration of structurally diverse drugs. *J. Pharm. Biomed. Anal.* **1997**, *15*, 469–477, doi:10.1016/s0731-7085(96)01883-3.
37. Kudo, S.; Umehara, K.; Abe, Y.; Furukawa, M.; Odomi, M. Intracerebral penetration of carteolol hydrochloride in rats. *Psychopharmacology (Berl.)* **1997**, *131*, 388–393, doi:10.1007/s002130050307.
38. Tsuneizumi, T.; Babb, S.M.; Cohen, B.M. Drug distribution between blood and brain as a determinant of antipsychotic drug effects. *Biol. Psychiatry* **1992**, *32*, 817–824, doi:10.1016/0006-3223(92)90085-e.
39. Young, R.C.; Mitchell, R.C.; Brown, T.H.; Ganellin, C.R.; Griffiths, R.; Jones, M.; Rana, K.K.; Saunders, D.; Smith, I.R.; Sore, N.E.; et al. Development of a new physicochemical model for brain penetration and its application to the design of centrally acting H2 receptor histamine antagonists. *J. Med. Chem.* **1988**, *31*, 656–671, doi:10.1021/jm00398a028.
40. Javaid, J.I.; Davis, J.M. Cocaine disposition in discrete regions of rat brain. *Biopharm. Drug Dispos.* **1993**, *14*, 357–364, doi:10.1002/bdd.2510140408.
41. Xie, R.; Hammarlund-Udenaes, M. Blood-brain barrier equilibration of codeine in rats studied with microdialysis. *Pharm. Res.* **1998**, *15*, 570–575, doi:10.1023/a:1011929910782.
42. Poulin, P.; Theil, F.P. A priori prediction of tissue:plasma partition coefficients of drugs to facilitate the use of physiologically-based pharmacokinetic models in drug discovery. *J. Pharm. Sci.* **2000**, *89*, 16–35, doi:10.1002/(SICI)1520-6017(200001)89:1<16::AID-JPS3>3.0.CO;2-E.
43. Csanády, G.A.; Oberste-Frielinghaus, H.R.; Semder, B.; Baur, C.; Schneider, K.T.; Filser, J.G. Distribution and unspecific protein binding of the xenoestrogens bisphenol A and daidzein. *Arch. Toxicol.* **2002**, *76*, 299–305, doi:10.1007/s00204-002-0339-5.
44. Platts, J.A.; Abraham, M.H.; Zhao, Y.H.; Hersey, A.; Ijaz, L.; Butina, D. Correlation and prediction of a large blood-brain distribution data set – an LFER study. *Eur. J. Med. Chem.* **2001**, *36*, 719–730, doi:10.1016/s0223-5234(01)01269-7.
45. Glynn, S.L.; Yazdanian, M. In vitro blood-brain barrier permeability of nevirapine compared to other HIV antiretroviral agents. *J. Pharm. Sci.* **1998**, *87*, 306–310, doi:10.1021/js970291i.
46. Mahar Doan, K.M.; Wring, S.A.; Shampine, L.J.; Jordan, K.H.; Bishop, J.P.; Kratz, J.; Yang, E.; Serabjit-Singh, C.J.; Adkison, K.K.; Polli, J.W. Steady-state brain concentrations of antihistamines in rats: interplay of membrane permeability, P-glycoprotein efflux and plasma protein binding. *Pharmacology* **2004**, *72*, 92–98, doi:10.1159/000079137.
47. Poulin, P.; Theil, F.-P. Prediction of pharmacokinetics prior to in vivo studies. II. Generic physiologically based pharmacokinetic models of drug disposition. *J. Pharm. Sci.* **2002**, *91*, 1358–1370, doi:10.1002/jps.10128.
48. Kalvass, J.C.; Maurer, T.S.; Pollack, G.M. Use of plasma and brain unbound fractions to assess the extent of brain distribution of 34 drugs: comparison of unbound concentration ratios to in vivo P-glycoprotein efflux ratios. *Drug Metab. Dispos.* **2007**, *35*, 660–666, doi:10.1124/dmd.106.012294.
49. Mitterhauser, M.; Wadsak, W.; Wabnegger, L.; Mien, L.-K.; Tögel, S.; Langer, O.; Sieghart, W.; Viernstein, H.; Kletter, K.; Dudczak, R. Biological evaluation of 2′-[18F]fluoroflumazenil ([18F]FFMZ), a potential GABA receptor ligand for PET. *Nucl. Med. Biol.* **2004**, *31*, 291–295, doi:10.1016/j.nucmedbio.2003.09.003.
50. Levêque, P.; Labar, D.; Gallez, B. Biodistribution, binding specificity and metabolism of [18F]fluoroethylflumazenil in rodents. *Nucl. Med. Biol.* **2001**, *28*, 809–814, doi:10.1016/s0969-8051(01)00251-7.

51. Wójcikowski, J.; Daniel, W.A. Thioridazine-fluoxetine interaction at the level of the distribution process in vivo. *Pol. J. Pharmacol.* **2002**, *54*, 647–654.
52. Gómez, L.E.; Cueva-Rolón, R.; Lehmann, P.A. Disposition kinetics of HEPP in rats after intravenous, oral, and intraperitoneal administration. Correlation of plasma and brain levels with the anticonvulsant effect. *Biopharm. Drug Dispos.* **1995**, *16*, 77–89, doi:10.1002/bdd.2510160203.
53. Waterhouse, R.N.; Gotsick, J.T.; Kabalka, G.W.; Goodman, M.M.; O'Brien, J.C. In vivo evaluation of [123I]-8-[4-[2-(5-iodothienyl)]-4-oxobutyl]-3-methyl-1-phenyl-1,3 ,8-tri azaspiro[4.5]decan-4-one as a potential dopamine D2 receptor radioligand for SPECT. *Nucl. Med. Biol.* **1998**, *25*, 77–80, doi:10.1016/s0969-8051(97)00137-6.
54. Waterhouse, R.N.; Mardon, K.; Giles, K.M.; Collier, T.L.; O'Brien, J.C. Halogenated 4-(phenoxymethyl)piperidines as potential radiolabeled probes for sigma-1 receptors: in vivo evaluation of [123I]-1-(iodopropen-2-yl)-4-[(4-cyanophenoxy)methyl]piperi dine. *J. Med. Chem.* **1997**, *40*, 1657–1667, doi:10.1021/jm960720+.
55. Yazdanian, M. Blood-brain barrier properties of human immunodeficiency virus antiretrovirals. *J. Pharm. Sci.* **1999**, *88*, 950–954, doi:10.1021/js990174i.
56. Castel-Branco, M.; Lebre, V.; Falcão, A.; Figueiredo, I.; Caramona, M. Relationship between plasma and brain levels and the anticonvulsant effect of lamotrigine in rats. *Eur. J. Pharmacol.* **2003**, *482*, 163–168, doi:10.1016/j.ejphar.2003.09.065.
57. Oldendorf, W.H.; Hyman, S.; Braun, L.; Oldendorf, S.Z. Blood-brain barrier: penetration of morphine, codeine, heroin, and methadone after carotid injection. *Science* **1972**, *178*, 984–986, doi:10.1126/science.178.4064.984.
58. Baudry, S.; Pham, Y.T.; Baune, B.; Vidrequin, S.; Crevoisier, C.; Gimenez, F.; Farinotti, R. Stereoselective passage of mefloquine through the blood-brain barrier in the rat. *J. Pharm. Pharmacol.* **1997**, *49*, 1086–1090, doi:10.1111/j.2042-7158.1997.tb06047.x.
59. Rivière, G.J.; Gentry, W.B.; Owens, S.M. Disposition of methamphetamine and its metabolite amphetamine in brain and other tissues in rats after intravenous administration. *J. Pharmacol. Exp. Ther.* **2000**, *292*, 1042–1047.
60. Aravagiri, M.; Teper, Y.; Marder, S.R. Pharmacokinetics and tissue distribution of olanzapine in rats. *Biopharm. Drug Dispos.* **1999**, *20*, 369–377, doi:10.1002/1099-081x(199911)20:8<369::aid-bdd200>3.0.co;2-6.
61. Raje, S.; Cao, J.; Newman, A.H.; Gao, H.; Eddington, N.D. Evaluation of the blood-brain barrier transport, population pharmacokinetics, and brain distribution of benztropine analogs and cocaine using in vitro and in vivo techniques. *J. Pharmacol. Exp. Ther.* **2003**, *307*, 801–808, doi:10.1124/jpet.103.053504.
62. Proksch, J.W.; Gentry, W.B.; Owens, S.M. The effect of rate of drug administration on the extent and time course of phencyclidine distribution in rat brain, testis, and serum. *Drug Metab. Dispos.* **2000**, *28*, 742–747.
63. Bickel, M.H.; Gerny, R. Drug distribution as a function of binding competition. Experiments with the distribution dialysis technique. *J. Pharm. Pharmacol.* **1980**, *32*, 669–674, doi:10.1111/j.2042-7158.1980.tb13035.x.
64. Doze, P.; Elsinga, P.H.; Maas, B.; Van Waarde, A.; Wegman, T.; Vaalburg, W. Synthesis and evaluation of radiolabeled antagonists for imaging of beta-adrenoceptors in the brain with PET. *Neurochem. Int.* **2002**, *40*, 145–155, doi:10.1016/s0197-0186(01)00081-x.
65. Wala, E.P.; Sloan, J.W.; Jing, X. Pharmacokinetics of the peripheral benzodiazepine receptor antagonist, PK 11195, in rats. The effect of dose and gender. *Pharmacol. Res.* **2000**, *41*, 461–468, doi:10.1006/phrs.1999.0617.
66. Dutta, S.; Matsumoto, Y.; Muramatsu, A.; Matsumoto, M.; Fukuoka, M.; Ebling, W.F. Steady-state propofol brain:plasma and brain:blood partition coefficients and the effect-site equilibration paradox. *Br. J. Anaesth.* **1998**, *81*, 422–424, doi:10.1093/bja/81.3.422.
67. Haddad, S.; Withey, J.; Laparé, S.; Law, F.; Krishnan, K. Physiologically-based pharmacokinetic modeling of pyrene in the rat. *Environ. Toxicol. Pharmacol.* **1998**, *5*, 245–255, doi:10.1016/s1382-6689(98)00008-8.
68. Lourenco, C.M.; Houle, S.; Wilson, A.A.; DaSilva, J.N. Characterization of R-[11C]rolipram for PET imaging of phosphodieterase-4: *in vivo* binding, metabolism, and dosimetry studies in rats. *Nucl. Med. Biol.* **2001**, *28*, 347–358, doi:10.1016/s0969-8051(01)00206-2.
69. Zhang, J.; McCarthy, T.J.; Moore, W.M.; Currie, M.G.; Welch, M.J. Synthesis and evaluation of two positron-labeled nitric oxide synthase inhibitors, S-[11C]methylisothiourea and S-(2-[18F]fluoroethyl)isothiourea, as potential positron emission tomography tracers. *J. Med. Chem.* **1996**, *39*, 5110–5118, doi:10.1021/jm960481q.
70. Caccia, S.; Fong, M.H. Kinetics and distribution of the beta-adrenergic agonist salbutamol in rat brain. *J. Pharm. Pharmacol.* **1984**, *36*, 200–202, doi:10.1111/j.2042-7158.1984.tb06941.x.
71. Washington, C.B.; Wiltshire, H.R.; Man, M.; Moy, T.; Harris, S.R.; Worth, E.; Weigl, P.; Liang, Z.; Hall, D.; Marriott, L.; et al. The disposition of saquinavir in normal and P-glycoprotein deficient mice, rats, and in cultured cells. *Drug Metab. Dispos.* **2000**, *28*, 1058–1062.
72. Hosseini-Yeganeh, M.; McLachlan, A.J. Tissue distribution of terbinafine in rats. *J. Pharm. Sci.* **2001**, *90*, 1817–1828, doi:10.1002/jps.1132.
73. Wilkinson, J.M.; Pollard, I. Accumulation of theophylline, theobromine and paraxanthine in the fetal rat brain following a single oral dose of caffeine. *Brain Res. Dev. Brain Res.* **1993**, *75*, 193–199, doi:10.1016/0165-3806(93)90023-4.
74. Rose, K.; Hall, L.H.; Kier, L.B. Modeling blood-brain barrier partitioning using the electrotopological state. *J. Chem. Inf. Comput. Sci.* **2002**, *42*, 651–666, doi:10.1021/ci010127n.
75. Yap, C.W.; Chen, Y.Z. Quantitative Structure-Pharmacokinetic Relationships for drug distribution properties by using general regression neural network. *J. Pharm. Sci.* **2005**, *94*, 153–168, doi:10.1002/jps.20232.
76. Narayanan, R.; Gunturi, S.B. In silico ADME modelling: prediction models for blood-brain barrier permeation using a systematic variable selection method. *Bioorg. Med. Chem.* **2005**, *13*, 3017–3028, doi:10.1016/j.bmc.2005.01.061.
77. Lin, J.H.; Chen, I.W.; Lin, T.H. Blood-brain barrier permeability and in vivo activity of partial agonists of benzodiazepine receptor: a study of L-663,581 and its metabolites in rats. *J. Pharmacol. Exp. Ther.* **1994**, *271*, 1197–1202.
78. Garg, P.; Verma, J. In silico prediction of blood brain barrier permeability: an Artificial Neural Network model. *J. Chem. Inf. Model.* **2006**, *46*, 289–297, doi:10.1021/ci050303i.

79. Luco, J.M. Prediction of the brain-blood distribution of a large set of drugs from structurally derived descriptors using partial least-squares (PLS) modeling. *J. Chem. Inf. Comput. Sci.* **1999**, *39*, 396–404, doi:10.1021/ci980411n.
80. Richardson, T.I.; Frank, S.A.; Wang, M.; Clarke, C.A.; Jones, S.A.; Ying, B.-P.; Kohlman, D.T.; Wallace, O.B.; Shepherd, T.A.; Dally, R.D.; et al. Structure-activity relationships of SERMs optimized for uterine antagonism and ovarian safety. *Bioorg. Med. Chem. Lett.* **2007**, *17*, 3544–3549, doi:10.1016/j.bmcl.2007.04.044.
81. Zhang, L.; Zhu, H.; Oprea, T.I.; Golbraikh, A.; Tropsha, A. QSAR modeling of the blood-brain barrier permeability for diverse organic compounds. *Pharm. Res.* **2008**, *25*, 1902–1914, doi:10.1007/s11095-008-9609-0.
82. Deák, K.; Takács-Novák, K.; Kapás, M.; Vastag, M.; Tihanyi, K.; Noszál, B. Physico-chemical characterization of a novel group of dopamine D(3)/D(2) receptor ligands, potential atypical antipsychotic agents. *J. Pharm. Biomed. Anal.* **2008**, *48*, 678–684, doi:10.1016/j.jpba.2008.06.021.
83. Hemmateenejad, B.; Miri, R.; Safarpour, M.A.; Mehdipour, A.R. Accurate prediction of the blood-brain partitioning of a large set of solutes using *ab initio* calculations and genetic neural network modeling. *J. Comput. Chem.* **2006**, *27*, 1125–1135, doi:10.1002/jcc.20437.
84. Hou, T.J.; Xu, X.J. ADME evaluation in drug discovery. 3. Modeling blood-brain barrier partitioning using simple molecular descriptors. *J. Chem. Inf. Comput. Sci.* **2003**, *43*, 2137–2152, doi:10.1021/ci034134i.
85. Muehlbacher, M.; Spitzer, G.M.; Liedl, K.R.; Kornhuber, J. Qualitative prediction of blood-brain barrier permeability on a large and refined dataset. *J. Comput.-Aided Mol. Des.* **2011**, *25*, 1095–1106, doi:10.1007/s10822-011-9478-1.
86. Polli, J.W.; Baughman, T.M.; Humphreys, J.E.; Jordan, K.H.; Mote, A.L.; Salisbury, J.A.; Tippin, T.K.; Serabjit-Singh, C.J. P-glycoprotein influences the brain concentrations of cetirizine (Zyrtec), a second-generation non-sedating antihistamine. *J. Pharm. Sci.* **2003**, *92*, 2082–2089, doi:10.1002/jps.10453.
87. Garberg, P.; Ball, M.; Borg, N.; Cecchelli, R.; Fenart, L.; Hurst, R.D.; Lindmark, T.; Mabondzo, A.; Nilsson, J.E.; Raub, T.J.; et al. In vitro models for the blood-brain barrier. *Toxicol. In Vitro* **2005**, *19*, 299–334, doi:10.1016/j.tiv.2004.06.011.
88. Zagol-Ikapitte, I.; Amarnath, V.; Jadhav, S.; Oates, J.A.; Boutaud, O. Determination of 3-methoxysalicylamine levels in mouse plasma and tissue by liquid chromatography-tandem mass spectrometry: application to *in vivo* pharmacokinetics studies. *J. Chromatogr. B. Analyt. Technol. Biomed. Life Sci.* **2011**, *879*, 1098–1104, doi:10.1016/j.jchromb.2011.03.026.
89. Winkler, D.A.; Burden, F.R. Modelling blood-brain barrier partitioning using Bayesian neural nets. *J. Mol. Graph. Model.* **2004**, *22*, 499–505, doi:10.1016/j.jmgm.2004.03.010.
90. Abraham, M.H.; Chadha, H.S.; Mitchell, R.C. Hydrogen bonding. 33. Factors that influence the distribution of solutes between blood and brain. *J. Pharm. Sci.* **1994**, *83*, 1257–1268, doi:10.1002/jps.2600830915.
91. Mente, S.R.; Lombardo, F. A recursive-partitioning model for blood-brain barrier permeation. *J. Comput.-Aided Mol. Des.* **2005**, *19*, 465–481, doi:10.1007/s10822-005-9001-7.
92. Van Damme, S.; Langenaeker, W.; Bultinck, P. Prediction of blood-brain partitioning: a model based on ab initio calculated quantum chemical descriptors. *J. Mol. Graph. Model.* **2008**, *26*, 1223–1236, doi:10.1016/j.jmgm.2007.11.004.
93. Bendels, S.; Kansy, M.; Wagner, B.; Huwyler, J. In silico prediction of brain and CSF permeation of small molecules using PLS regression models. *Eur. J. Med. Chem.* **2008**, *43*, 1581–1592, doi:10.1016/j.ejmech.2007.11.011.
94. Timmermans, P.B.; Brands, A.; van Zwieten, P.A. Lipophilicity and brain disposition of clonidine and structurally related imidazolidines. *Naunyn Schmiedebergs Arch. Pharmacol.* **1977**, *300*, 217–226, doi:10.1007/BF00500963.
95. Perleberg, U.R.; Keys, D.A.; Fisher, J.W. Development of a physiologically based pharmacokinetic model for decane, a constituent of jet propellent-8. *Inhal. Toxicol.* **2004**, *16*, 771–783, doi:10.1080/08958370490490473.
96. Konovalov, D.A.; Coomans, D.; Deconinck, E.; Heyden, Y.V. Benchmarking of QSAR models for blood-brain barrier permeation. *J. Chem. Inf. Model.* **2007**, *47*, 1648–1656, doi:10.1021/ci700100f.
97. West, D.C.; Qin, Y.; Peterson, Q.P.; Thomas, D.L.; Palchaudhuri, R.; Morrison, K.C.; Lucas, P.W.; Palmer, A.E.; Fan, T.M.; Hergenrother, P.J. Differential effects of procaspase-3 activating compounds in the induction of cancer cell death. *Mol Pharm* **2012**, *9*, 1425–1434, doi:10.1021/mp200673n.
98. García-Osta, A.; Cuadrado-Tejedor, M.; García-Barroso, C.; Oyarzábal, J.; Franco, R. Phosphodiesterases as therapeutic targets for Alzheimer's disease. *ACS Chem. Neurosci.* **2012**, *3*, 832–844, doi:10.1021/cn3000907.
99. Yeh, H.-H.; Tian, M.; Hinz, R.; Young, D.; Shavrin, A.; Mukhapadhyay, U.; Flores, L.G.; Balatoni, J.; Soghomonyan, S.; Jeong, H.J.; et al. Imaging epigenetic regulation by histone deacetylases in the brain using PET/MRI with ^{18}F-FAHA. *Neuroimage* **2013**, *64*, 630–639, doi:10.1016/j.neuroimage.2012.09.019.
100. Bricker, B.; Jackson, T.; Boateng, B.; Zhu, X.Y.; Ablordeppey, S.Y. Evaluation of the behavioral and pharmacokinetic profile of SYA013, a homopiperazine analog of haloperidol in rats. *Pharmacol. Biochem. Behav.* **2012**, *102*, 294–301, doi:10.1016/j.pbb.2012.05.003.
101. Lee, S.; Zheng, X.; Krishnamoorthy, J.; Savelieff, M.G.; Park, H.M.; Brender, J.R.; Kim, J.H.; Derrick, J.S.; Kochi, A.; Lee, H.J.; et al. Rational design of a structural framework with potential use to develop chemical reagents that target and modulate multiple facets of Alzheimer's disease. *J. Am. Chem. Soc.* **2014**, *136*, 299–310, doi:10.1021/ja409801p.
102. Seo, Y.J.; Kang, Y.; Muench, L.; Reid, A.; Caesar, S.; Jean, L.; Wagner, F.; Holson, E.; Haggarty, S.J.; Weiss, P.; et al. Image-guided synthesis reveals potent blood-brain barrier permeable histone deacetylase inhibitors. *ACS Chem. Neurosci.* **2014**, *5*, 588–596, doi:10.1021/cn500021p.
103. Ganesh, T.; Jiang, J.; Yang, M.-S.; Dingledine, R. Lead optimization studies of cinnamic amide EP2 antagonists. *J. Med. Chem.* **2014**, *57*, 4173–4184, doi:10.1021/jm5000672.
